# Nomograms for predicting overall survival and cancer-specific survival in patients with head and neck non-Hodgkin lymphoma: A population-based study

**DOI:** 10.1097/MD.0000000000032865

**Published:** 2023-02-10

**Authors:** Jing Peng, Jianming Chen, Yucheng Liu, Jun Lyu, Bin Zhang

**Affiliations:** a Department of Orthodontics, Affiliated Stomatology Hospital of Guangzhou Medical University, Guangdong Engineering Research Center of Oral Restoration and Reconstruction, Guangzhou Key Laboratory of Basic and Applied Research of Oral Regenerative Medicine, Guangzhou, Guangdong, China; b Department of Clinical Research, The First Affiliated Hospital of Jinan University, Guangzhou, Guangdong, China.

**Keywords:** cancer-specific survival, head and neck non-Hodgkin lymphoma, nomogram, overall survival, prognosis

## Abstract

This study aimed to develop comprehensive nomograms for patients with head and neck non-Hodgkin lymphoma (H&NNHL) to determine their overall survival (OS) and cancer-specific survival (CSS). In this study, 602 H&NNHL patients were analyzed from the Surveillance, Epidemiology, and End Results database. The R software was used to randomly divide the patients into the training cohort (n = 421) and the validation cohort (n = 181) in a 7-to-3 ratio. To develop nomograms for projecting OS and CSS, multivariable Cox regression was used to acquire independent predictive factors. We have constructed nomograms to predict the 3-, 5-, and 8-year OS and CSS probabilities of H&NNHL patients. The consistency index of the nomograms for OS (CSS) was 0.74 (0.778) and 0.734 (0.775), in the training and validation cohort respectively, and was higher than that of the Ann Arbor staging system. Calibration plotting showed that our models have good calibration ability. Moreover, assessments of the area under the time-dependent receiver operating characteristics curve, net reclassification improvement, integrated discrimination improvement and decision curve analysis demonstrated that our nomograms performed better and were more clinically useful than the Ann Arbor staging system. This is the first research to establish comprehensive nomograms for predicting OS and CSS in patients with H&NNHL at 3-, 5-, and 8-year. The validation of the models demonstrated good performance. It can provide clinicians with reference information for determining customized clinical treatment options and providing personalized prognoses.

Indexes such as the concordance index, the area under the time-dependent receiver operating characteristics curve, calibration curves, the net reclassification improvement, the integrated discrimination improvement, and decision-curve analysis were used to compare new survival models to the classical Ann Arbor staging system.

## 1. Introduction

In the area of the head and neck, lymphoma is the third largest malignant tumor after squamous cell carcinoma and thyroid cancer.^[1,2]^ However, non-Hodgkin lymphoma (NHL) makes up 90.15% of lymphoma and is the sixth most common cause of cancer-related death in the USA.^[3]^ In the latest World Health Organization classification, NHL has >50 different subtypes and can occur throughout the body; hence, the treatment and prognosis are different and complicated.^[4]^ The Ann Arbor staging system has been commonly used for staging and prognostic evaluation of the NHL, consistent with the American Joint Committee on Cancer (AJCC) 7th staging system.^[5]^ Nevertheless, several other vital characteristics such as gender, marital status, social-economic status, histological subtypes, and treatment options may also affect the survival of each patient. Nomogram is typically used as a prognostic tool to assist clinicians in accurately estimating the personalized prognosis. Nevertheless, to the best of our knowledge, no nomogram has been reported for patients with head and neck non-Hodgkin lymphoma (H&NNHL). Thus, the current study focused on constructing and validating nomograms for predicting overall survival (OS) and cancer-specific survival (CSS) based on the Surveillance, Epidemiology, and End Results (SEER) database, and the performance of new prediction models was compared with that of the Ann Arbor staging system.

## 2. Materials

The study population was extracted from the SEER database of the United States National Cancer Institute using its software SEER*Stat 8.4.0.1 (seer.cancer.gov/seerstat/), which is a collection of 17 population-based cancer registries covering around 26.5% of the USA population. Informed consent for patient information to be published in this article was not obtained because data for this research are available from SEER database (https://seer.cancer.gov/). A retrospective search was performed for cases diagnosed with H&NNHL between 2000 and 2015. Briefly, cases selected through The Third edition of the International Classification of Diseases for Oncology histological type codes: 9591-3, 9596-3, 9712-3, 9714-3, 9715-3, 9719-3, and the topography codes: C00.0-C14.8. Patients were excluded due to unknown Ann Arbor stage and ambiguous pathological diagnosis.

Available data included age at diagnosis, gender, race, marital status, income, residence information, sequence number, primary site, histology type, tumor stage, laterality, Ann Arbor stage, B symptoms, nodal status, systemic treatment, surgery therapy, radiotherapy, chemotherapy, survival months, vital status, and cause of death. The main outcomes of interest were OS and CSS. We define OS as the time from diagnosis to death attributed to any cause, whereas CSS is defined as the length of time from the diagnosis to the death that was attributable to H&NNHL.

We employed SPSS software (version 24.0, Chicago, IL) and R-software (version 4.2.1, www.r-project.org) to perform the statistical analyses. Statistical significance was determined by a bilateral *P* value of <.05. We conducted a descriptive analysis of all of the mentioned factors. The continuous variable was expressed as median (25th–75th percentile) values and the categorical variable was expressed as percentages. R-software grouped patients into the training and validation cohorts in a 7:3 ratio.

Multivariable analysis using the Cox proportional hazards regression model was performed to identify significant independent prognostic factors in the training cohort, for which *P* = .1. Nomograms for prediction of 3-, 5-, 8-year OS and CSS probability were constructed by utilizing the selected factors, and hazard ratios (HRs) with 95% confidence intervals were reported. The consistency index and the area under the time-dependent receiver operating characteristics curve (AUC) were used to assess the discriminability and accuracy of the nomograms, compared with the Ann Arbor staging system.^[6]^ Then 2 relatively new indicators (net reclassification improvement [NRI] and integrated discrimination improvement [IDI]) were supplemented to increase the accuracy and comprehensiveness of the comparisons.^[7,8]^ To assess the consistency between anticipated and detected survival, the calibration curves of the nomograms generated by bootstrapping with 500 resample were utilized. Furthermore, decision curve analyses were employed to evaluate the clinical effectiveness of the nomograms.^[9]^

## 3. Results

### 3.1. Patient characteristics

**Figure 1.**
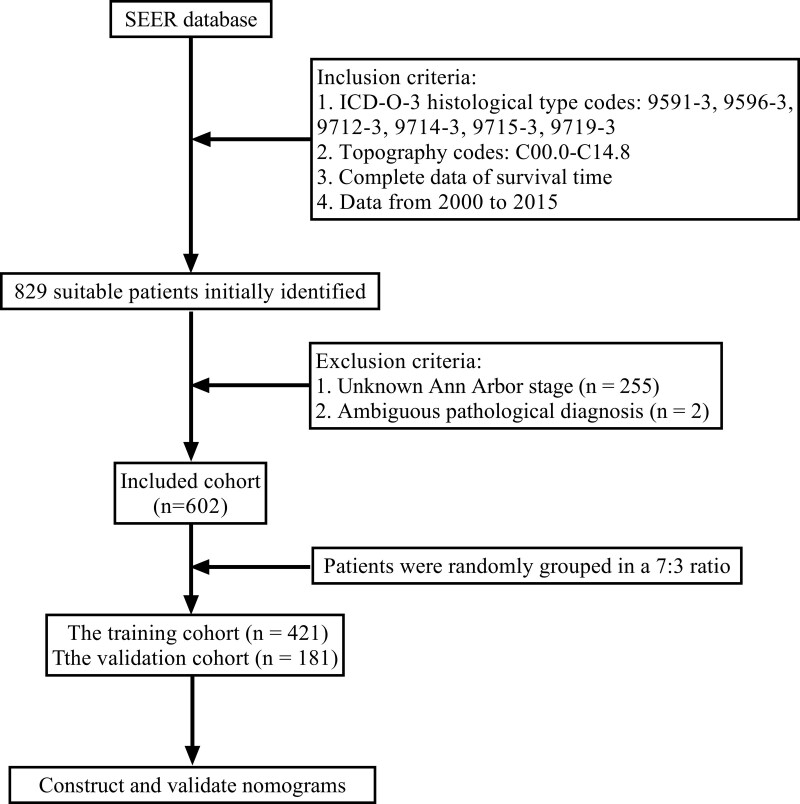
Research flowchart.

Totally, 829 suitable patients were initially identified from the SEER database, they were carefully screened and finally, 602 patients with H&NNHL were included in this study (255 patients were excluded due to unknown Ann Arbor stage, with further 2 patients rejected due to ambiguous pathological diagnosis [Fig. 1]). They were divided randomly into 2 parts: the training cohort (n = 421) and the validation cohort (n = 181). Patients’ median age at diagnosis was 65.0 (49.0, 79.0) and the majority of them were male (55.0%), white (80.7%), and unmarried (55.0%). Most patients lived in metropolitan countries (87.7%) and the most common level of household income was between $55,000 to $74,999 (47.5%). Among the tumor-related features, 82.1% of the tumors occupied the primary sequence and the most primary site was salivary glands (30.4%). Ann Arbor I (48.3%) was the most frequent stage and most of the histology type was not otherwise specified (NOS, 70.6%). Besides, only 19.8% of tumors occurred in the nodal and paired sides (3.5%). Furthermore, in most cases, patients did not have any systematic symptoms (91.9%). Regarding the therapeutic strategies, more than half of the patients underwent chemotherapy (55.8%), while most of them did not accept systemic therapy (90.2%), surgery (72.3%), and radiotherapy (60.8%).

The median survival time was 62 months, 27.4% of patients died from H&NNHL itself and the mortality of overall data was 58.8%. The socio-demographic and clinicopathologic characteristics of the patients are presented in Table 1, and there was no statistical difference between the validation cohort and the training cohort.

**Table 1:** Socio-demographic and clinicopathologic characteristics of 602 eligible patients.

Variable	Validation cohort (n = 181)	Training cohort (n = 421)	Overall (n = 602)	*P* value
Age at diagnosing	66.0 (48.0, 79.0)	65.0 (50.0, 80.0)	65.0 (49.0, 79.0)	.968
Gender (%)				.724
Male	102 (56.4)	229 (54.4)	331 (55.0)	
Female	79 (43.6)	192 (45.6)	271 (45.0)	
Race (%)				.067
White	145 (80.1)	341 (81.0)	486 (80.7)	
Black	18 (9.9)	23 (5.5)	41 (6.8)	
Asian or Pacific Islander	18 (9.9)	50 (11.9)	68 (11.3)	
Others/Unknown	0 (0.0)	7 (1.7)	7 (1.2)	
Marital status (%)				.59
Married	84 (46.4)	187 (44.4)	271 (45.0)	
Unmarried	97 (53.6)	234 (55.6)	331 (55.0)	
Income (%)				.997
<$35,000	2 (1.1)	5 (1.2)	7 (1.2)	
$35,000–$54,999	35 (19.3)	81 (19.2)	116 (19.3)	
$55,000–$74,999	85 (47.0)	201 (47.7)	286 (47.5)	
> $75,000	59 (32.6)	134 (31.8)	193 (32.1)	
Residence information (%)				.776
Metropolitan	158 (87.3)	370 (87.9)	528 (87.7)	
Nonmetropolitan	23 (12.7)	50 (11.9)	73 (12.1)	
Unknown	0 (0.0)	1 (0.2)	1 (0.2)	
Sequence number (%)				.638
Primary	146 (80.7)	348 (82.7)	494 (82.1)	
Secondary	35 (19.3)	73 (17.3)	108 (17.9)	
Primary site (%)				.072
Oral cavity	34 (18.8)	96 (22.8)	130 (21.6)	
Salivary glands	57 (31.5)	126 (29.9)	183 (30.4)	
Pharynx	57 (31.5)	92 (21.9)	149 (24.8)	
Tonsil	29 (16.0)	94 (22.3)	123 (20.4)	
Overlapping lesion	4 (2.2)	13 (3.1)	17 (2.8)	
Histology type (%)				.349
NOS	131 (72.4)	294 (69.8)	425 (70.6)	
B cell	6 (3.3)	6 (1.4)	12 (2.0)	
ALK	11 (6.1)	32 (7.6)	43 (7.1)	
NKT	38 (21.0)	84 (20.0)	122 (20.3)	
Tumor stage (%)				.403
Distant	35 (19.3)	76 (18.1)	111 (18.4)	
Localized	93 (51.4)	198 (47.0)	291 (48.3)	
Regional	53 (29.3)	147 (34.9)	200 (33.2)	
Laterality (%)				1
One side	175 (96.7)	406 (96.4)	581 (96.5)	
Paired side	6 (3.3)	15 (3.6)	21 (3.5)	
B symptoms (%)				.803
B symptom(s)	16 (8.8)	33 (7.8)	49 (8.1)	
No B symptoms/Blank(s)	165 (91.2)	388 (92.2)	553 (91.9)	
Nodal status (%)				.34
Extranodal	150 (82.9)	333 (79.1)	483 (80.2)	
Nodal	31 (17.1)	88 (20.9)	119 (19.8)	
Ann Arbor stage (%)				.266
I	93 (51.4)	198 (47.0)	291 (48.3)	
II	53 (29.3)	147 (34.9)	200 (33.2)	
III	15 (8.3)	22 (5.2)	37 (6.1)	
IV	20 (11.0)	54 (12.8)	74 (12.3)	
Systemic treatment (%)				.261
Systemic therapy	22 (12.2)	37 (8.8)	59 (9.8)	
No systemic therapy	159 (87.8)	384 (91.2)	543 (90.2)	
Surgery therapy (%)				.798
Yes	52 (28.7)	115 (27.3)	167 (27.7)	
No/Unknown	129 (71.3)	306 (72.7)	435 (72.3)	
Radiotherapy (%)				.175
Yes	63 (34.8)	173 (41.1)	236 (39.2)	
No/Unknown	118 (65.2)	248 (58.9)	366 (60.8)	
Chemotherapy (%)				.178
Yes	93 (51.4)	243 (57.7)	336 (55.8)	
No/Unknown	88 (48.6)	178 (42.3)	266 (44.2)	

### 3.2. Variable screening and nomogram establishment

The multivariable analysis of OS revealed that the following factors were statistically significant: age at diagnosis (HR = 1.034, *P* < .001), being female (HR = 0.792, *P* < .1 versus male), being unmarried (HR = 1.355, *P* < .05 vs married), secondary site (HR = 1.379, *P* < .1 vs primary site), NK/T cell lymphoma (HR = 4.764, *P* < .001 vs NOS), Ann Arbor stage II (HR = 1.322, *P* < .1 vs stage I), Ann Arbor stage III (HR = 1.676, *P* < .1 vs stage I), Ann Arbor stage IV (HR = 1.717, *P* < .05 vs stage I), not receiving systemic therapy (HR = 2.065, *P* < .05 vs systemic therapy), no/unknown surgery status (HR = 1.411, *P* < .1 vs surgery), and no/unknown radiotherapy status (HR = 1.311, *P* < .05 vs radiotherapy).

The multivariable analysis of CSS demonstrated these factors were statistically significant: age at diagnosis (HR = 1.030, *P* < .001), unmarried (HR = 1.368, *P* < .1 vs married), salivary gland (HR = 0.521, *P* < .05 vs oral cavity), overlapping lesion (HR = 2.06, *P* < .1 vs oral cavity), NK/T cell lymphoma (HR = 5.300, *P* < .001 vs NOS), Ann Arbor stage II (HR = 1.468, *P* < .1 vs stage I), Ann Arbor stage III (HR = 2.347, *P* < .05 vs stage I), Ann stage IV (HR = 2.239, *P* < .05 vs stage I), not receiving radiotherapy (HR = 1.453, *P* < .1 vs radiotherapy). Tables 2 and 3 list the results of the multivariable Cox regression analysis of OS and CSS.

**Table 2:** Selected factors by multivariable Cox regression analysis of OS.

Variable	Multivariable analysis		
	HR	95% CI	*P* value
Age at diagnose	1.034	1.026–1.043	.000***
Gender			
Male	Reference		
Female	0.792	0.606–1.035	.088*
Marital status			
Married	Reference		
Unmarried	1.355	1.043–1.760	.023**
Sequence number			
Primary	Reference		
Secondary	1.379	0.998–1.906	.052*
Histology type			
NOS	Reference		
B cell	1.385	0.604–3.176	.441
ALK	0.996	0.584–1.801	.989
NKT	4.764	3.393–6.688	.000***
Ann Arbor stage			
I	Reference		
II	1.322	0.992–1.762	.056*
III	1.676	0.976–2.879	.061*
IV	1.717	1.173–2.515	.005**
Systemic treatment			
Systemic therapy	Reference		
No systemic therapy	2.065	1.016–4.199	.045**
Surgery therapy			
Yes	Reference		
No/Unknown	1.411	0.100–1.993	.050**
Radiotherapy			
Yes	Reference		
No/Unknown	1.311	1.002–1.717	.048**

CI = confidence interval, HR = hazard ratio, OS = overall survival.

* *P* < .1;

** *P* < .05;

*** *P* < .001.

**Table 3:** Selected factors by multivariable Cox regression analysis of CSS.

Variable	Multivariable analysis		
	HR	95% CI	*P* value
Age at diagnose	1.035	1.022–1.049	.000***
Marital status			
Married	Reference		
Unmarried	1.368	0.937–1.996	.093*
Primary site			
Oral cavity	Reference		
Salivary glands	0.521	0.293–0.929	.027**
Pharynx	0.881	0.535–1.450	.618
Tonsil	1.008	0.565–1.802	.977
Overlapping lesion	2.067	0.805–5.306	.131
Histology type			
NOS	Reference		
B cell	1.972	0.265–14.687	.508
ALK	1.570	0.733–3.359	.246
NKT	5.300	3.205–8.765	.000***
Ann Arbor stage			
I	Reference		
II	1.468	0.930–2.318	.099*
III	2.347	1.224–4.500	.010**
IV	2.239	1.352–3.707	.002**
Radiotherapy			
Yes	Reference		
No/Unknown	1.453	0.972–2.173	.068*

CI = confidence interval, CSS = cancer-specific survival, HR = hazard ratio.

* *P* < .1;

** *P* < .05;

*** *P* < .001.

**Figure 2.**
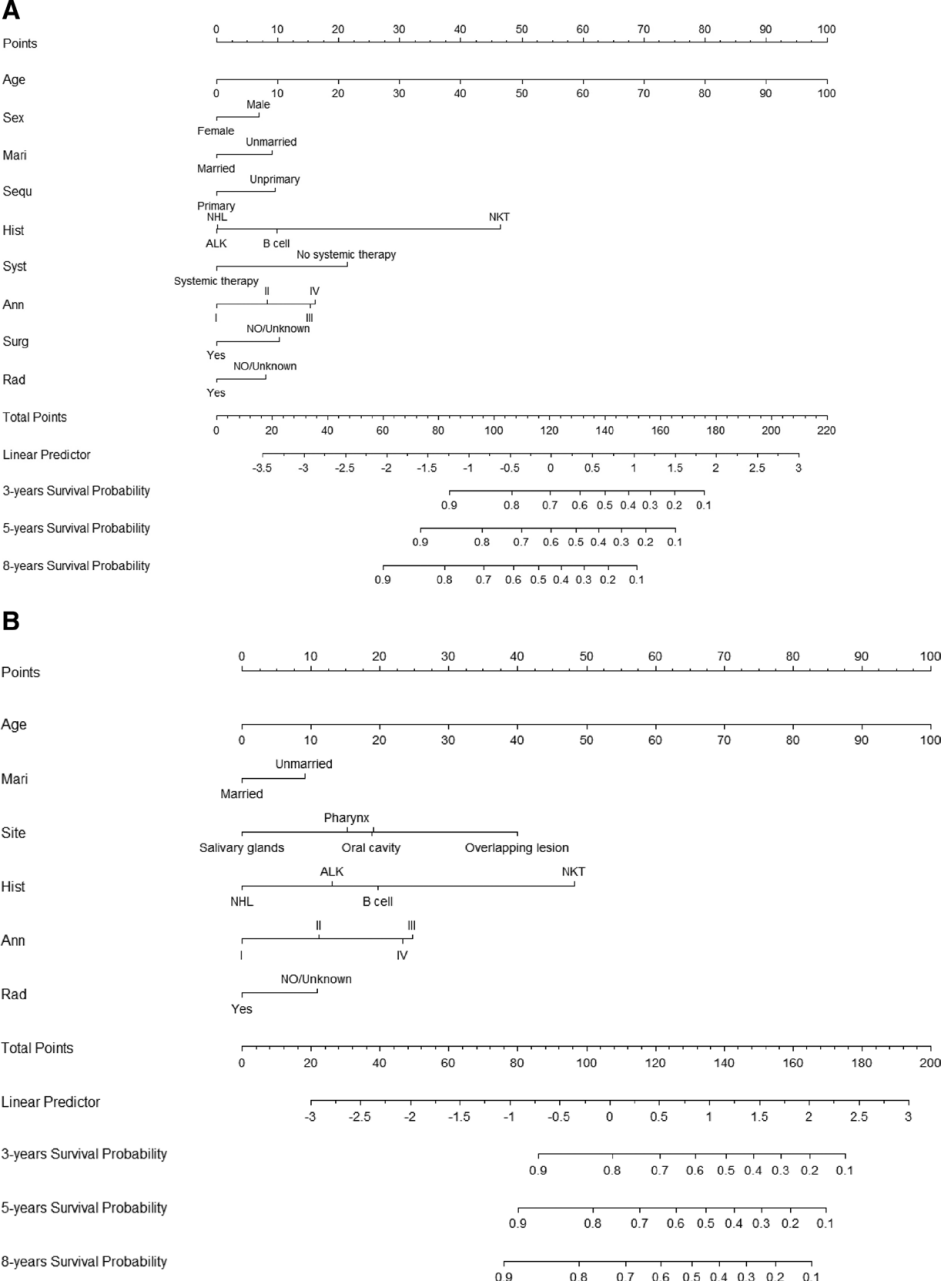
Nomogram predicting 3-, 5-, 8-year (A) OS probability and (B) CSS probability. Age = age at diagnosis, Ann = Ann Arbor stage, CSS = cancer-specific survival, Hist = histology type, Mari = marital status, OS = overall survival, Rad = radiotherapy Sequ = sequence number, Sex = gender, Site = primary site, Surg = surgery therapy, Syst = systemic treatment.

Figure 2A shows the nomogram predicting the 3-, 5-, and 8-year OS probabilities in H&NNHL patients. As can be seen, age at diagnosis had the greatest impact on prognosis, followed by histology type, the status of systemic therapy, Ann Arbor stage, surgery status, sequence number, marital status, radiotherapy status, and finally gender.

Nomogram to predict the 3-, 5-, and 8-year CSS rates was developed using the same method (Fig. 2B). Also, the age at diagnosis was the strongest significant factor, followed by histology type, primary site, Ann Arbor stage, radiotherapy status, and finally marital status.

### 3.3. Nomogram comparison and evaluation

**Figure 3.**
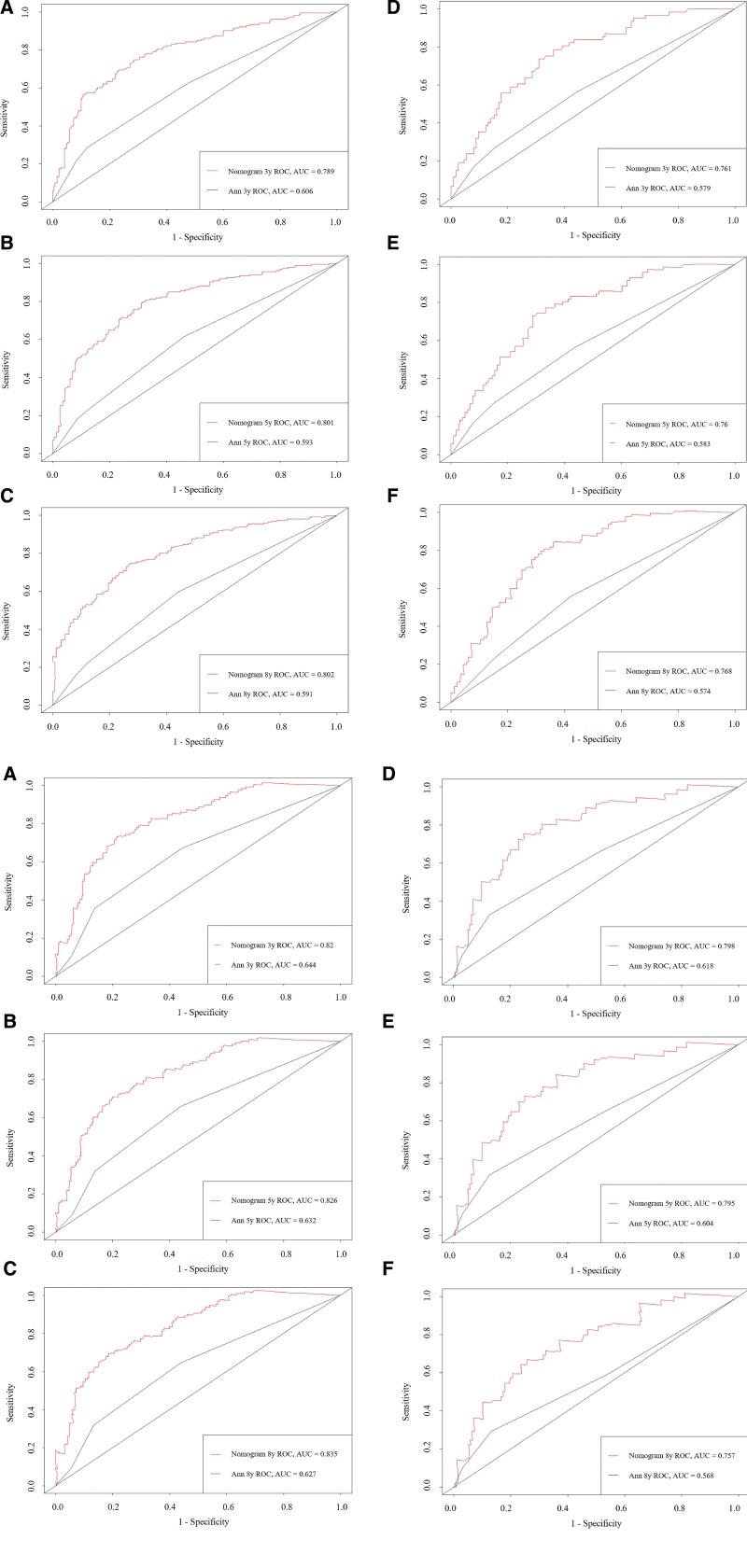
The area under the ROC curve (AUC) for (A) OS probability and (B) CSS probability. (A–C) The 3-, 5-, 8-year results of the training cohort. (D–F) The 3-, 5-, 8-year results of the validation cohort. CSS = cancer-specific survival, OS = overall survival, ROC = receiver operating characteristics curve.

**Figure 4.**
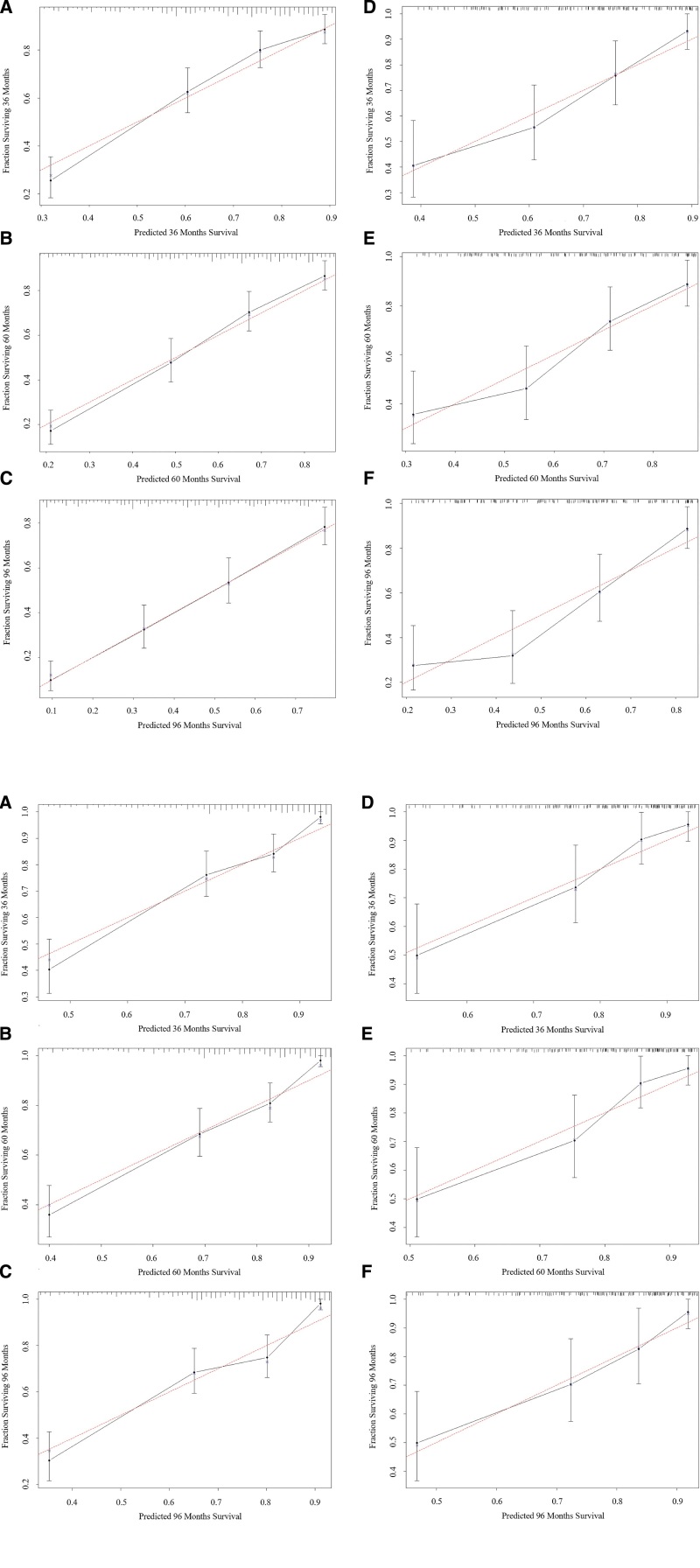
Calibration curves for (A) OS and (B) CSS probability depict the consistency between our calibration curves and the ideal lines. (A–C) The 3-, 5-, 8-year results of the training cohort. (D–F) The 3-, 5-, 8-year result of the validation cohort. CSS = cancer-specific survival, OS = overall survival.

**Figure 5.**
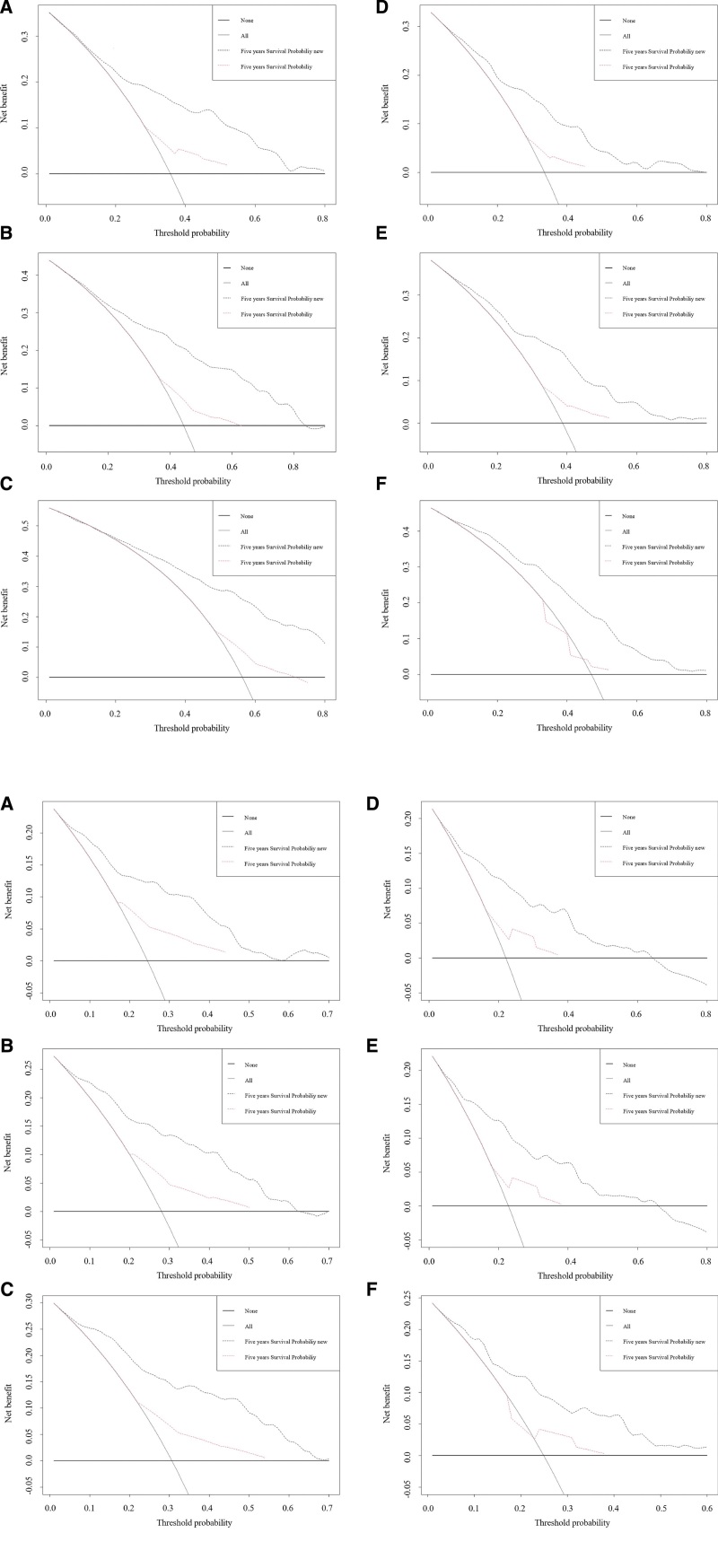
Decision curve analysis curves (DCA) for (A) OS and (B) CSS probability. (A–C) The 3-, 5-, 8-year results of the training cohort. (D–F) The 3-, 5-, 8-year results of the validation cohort. CSS = cancer-specific survival, OS = overall survival.

Once the nomograms were established, we used a series of indicators to evaluate the performance of the new prediction models. The consistency index for OS (CSS) prediction of the training and validation cohort were 0.74 (0.778) and 0.734 (0.775), which were higher than that of the Ann Arbor staging system (training cohort: 0.576 [0.611], validation cohort: 0.565 [0.598]). We further compared receiver operating characteristics curve curves for the OS (CSS) nomogram, and the 3-, 5-, 8-year AUC values in the training cohort were 0.789 (0.820), 0.801 (0.826), 0.802 (0.835), and 0.761 (0.798), 0.76 (0.795), 0.788 (0.757) in the validation cohort, which were higher than those of the old model too (Fig. 3A and B). The NRI values for the 3-, 5-, 8-year OS (CSS) probabilities were 0.764 (0.776), 0.857 (0.799), 0.904 (0.863) in the training cohort, and 0.711 (0.757), 0.763 (0.813), 0.827 (0.770) in the validation cohort. In addition, the IDI values for the 3-, 5-, 8-year OS (CSS) probabilities were 0.188 (0.135), 0.213 (0.149), 0.232 (0.159) in the training cohort, and 0.159 (0.124), 0.210 (0.163), 0.231 (0.172) in the validation cohort (*P* < .001). The calibration plots demonstrated that the standard curves of the 3-, 5-, 8-year OS (CSS) probabilities of the model had good calibration ability (Fig. 4A and B). Our nomograms proceeded net benefits in both the training and validation cohorts, as the 3-, 5-, and 8-year DCA curves displayed (Fig. 5A and B).

## 4. Discussion

Tumors in the area of the head and neck can be easily noticed and accessed, but the clinical behavior and manifestations of NHL usually lack specific characteristics without biopsy and histological evidence. Hence, the diagnosis of NHL should be taken into consideration by physicians in cases of unknown cervical or oral masses. H&NNHL is a lymphoproliferative malignancy, due to the great heterogeneity and inconsistency, treatment options and survival outcomes varied from patient to patient. Some researchers have reported prognostic factors of lymphoma in some local regions, such as the tongue, thyroid, laryngeal, and sinonasal.^[10–13]^ However, there is insufficient information on the region of the head and neck as a whole. Therefore, we developed and authenticated OS and CSS nomograms based on clinical characteristics and treatment approaches of patients with H&NNHL. In our nomograms, the C‐index and AUC exceeded 0.7, and NRI and IDI surpassed 0, showing that our new models performed better in discrimination performance and predictive ability, compared with the Ann Arbor staging system. The calibration plots showed excellent consistency between our calibration curves and the ideal lines. Also, the DCA curves of our new nomograms were all above those of the old model, which supported that our new models have better clinical effectiveness.

### 4.1. Age, gender, and marital status

We identified age at diagnosis as the highest score in both OS and CSS nomograms through the multivariate Cox regression analysis, which indicated that older patients have poorer survival. It’s in accordance with other research, considering the severity of frank pathologic dysfunction or comorbidity increases with age.^[14,15]^ According to our study, being unmarried is a risk factor for survival too (OS: HR = 1.355, *P* < .05, CSS: HR = 1.368, *P* < .05). We wouldn’t be surprised because some researchers even proved that the survival benefit associated with marriage was greater than the survival advantage published from chemotherapy for head and neck cancers. Our study also showed that in the nomogram for OS prediction, marital status had a higher weight than radiotherapy. Reasons may be that married patients could get better care and more encouragement to seek and accept prescribed treatments. Meanwhile, they may feel less distress, depression, and anxiety.^[16]^ Such achievements may suggest a psychosocial support-based intervention is warranted to prolong the OS of cancer patients.^[17]^ Moreover, male patients tend to have a worse prognosis than female patients in OS rather than CSS (HR = 0.792, *P* < .1, being female vs being male), it may due to the negative effects of smoking and alcohol intake rather than the disease itself.^[18]^

### 4.2. Ann Arbor stage

Our research showed a predominance of patients with an Ann Arbor Stage I diagnosis (48.3%), which may be attributed to a lower screening threshold and advanced detection in recent years.^[19]^ Generally speaking, the higher the Ann Arbor stage, the more invasive and widespread the condition. That is why the higher Ann Arbor stage often has been associated with more detrimental effects on patient survival and prognosis, as shown in our OS nomogram. Interestingly, to the best of our knowledge, our study revealed for the first time that stage III had a worse prognosis than stage IV for the CSS nomogram of patients with H&NNHL. It may support the evidence for the revision of the AJCC 8th staging system, as it restaged the status of NHL that any extra lymphatic involvement with nodal disease above and below the diaphragm from stage III to IV.^[20]^ Furthermore, the score of the Ann Arbor stage in predicting prognosis is lower than the age at diagnosis, histology type, primary site, and systematic therapy, indicating that only using it for prognosis evaluation of H&NNHL is limited and less effective.

### 4.3. Histology type, primary site, and sequence number

Histology type is another independent predictor for both CSS and OS. In our database, extranodal NK/T cell lymphoma accounted for 20.3% while having the worst prognosis, which is consistent with other research that it’s a rare and aggressive disease characterized by a high recurrence rate and poor prognosis.^[21]^ Besides, we classified the primary site of NHL as the oral cavity (21.6%), salivary glands (30.4%), pharynx (24.8%), tonsil (20.4%) and overlapping lesion (2.8%), it’s an independent predictive factor of CSS but not OS. However, the overlapping lesion had the lowest incidence but the worst prognosis. In addition, the secondary tumor had a worse prognosis than the primary tumor in OS rather than CSS, since it could have metastasized from other tumors.^[22]^ It should arouse enough attention of clinicians when they find a patient with secondary H&NNHL, occurring in the overlapping lesion with the histology type of extranodal NK/T cell lymphoma.

### 4.4. Treatment

Although there is a large variety of standard treatment strategies for H&NNHL, the internationally recognized treatment is chemotherapy, especially for diffuse large B-cell lymphoma (the commonest high-grade lymphoma) and ALK-negative anaplastic large-cell lymphoma.^[23,24]^ In contrast, patient with other types is not suitable for chemotherapy. For example, indolent lymphomas (mostly follicular lymphoma) may be cured by surgical excision or radiotherapy,^[23]^ radiation therapy alone is an effective therapy for early-stage NK/T cell lymphoma with no benefit of adding chemotherapy, and persons with advanced NK/T cell lymphoma could benefit from combined radiation therapy and chemotherapy.^[24]^ Our database showed that chemotherapy (55.8%) was the most common treatment, followed by radiotherapy (39.2%), surgery (27.7%), and systemic treatment (9.8%). Nevertheless, chemotherapy was not an independent factor in improving patients’ outcomes, whereas radiotherapy was. These findings were consistent with Shotaro’s conclusion that in the modern era, radiotherapy remains one of the most powerful and effective single-agent therapeutics for both aggressive and indolent NHL subtypes.^[25]^ In spite of this fact, the utilization of radiotherapy for aggressive NHL has been steadily decreasing, the reason is probably multifactorial, but a significant contributor may be the lasting concerns regarding the late toxicity profile, including the risk of cardiac mortality, endocrine dysfunction, or perhaps secondary malignancy.^[26–28]^ In addition, further research has shown that the incidence of cardiotoxicity is increasing among patients undergoing anthracycline-based chemotherapy, especially for the elderly, frail, or those with cardiac comorbidities. Hence, alternative treatment options should be tailored to the patient’s health status to achieve a maximal benefit-risk ratio.^[29]^ Our database was collected from 2000 to 2015, cases diagnosed nowadays are more likely to be treated with systemic therapy alone, combined modality therapy of systemic chemoimmunotherapy followed by consolidation radiotherapy.^[30]^ Our nomogram also showed that systemic treatment and surgery were significant to patients’ OS. Besides, new treatment strategies such as targeted blocking of molecular or cellular mechanisms and T-cell therapeutics may improve the clinical practice of NHL.^[31,32]^

### 4.5. Other scoring systems

In addition to the Ann Arbor staging system, there are other scoring systems used to evaluate the prognosis of NHL, one of which is the International Prognostic Index (IPI). IPI contains 5 prognostic factors: age >60 years, serum lactate dehydrogenase (LDH), Ann Arbor stage III/IV, the Eastern Cooperative Oncology Group performance status >2, and >1 site with extranodal involvement. It also categorizes patients into 4 risk groups: low-risk, low-intermediate risk, high-intermediate risk, and high-risk.^[33]^ However, Savage found that IPI can effectively distinguish low-risk and medium-low-risk groups, but for another group with >3 scores, it didn’t seem to work well.^[34]^ A study focused on 193 NHL patients showed that only a few patients had slightly elevated LDH levels, but the survival outcome did exist significant differences among patients.^[35]^ Our findings also revealed that though 80.2% of H&NNHL occurred in the extranodal setting, nodal status was not a significant prognostic factor in the OS and CSS nomogram, reinforcing the assumption that we cannot identify H&NNHL solely by laboratory findings.

### 4.6. Limitations

There are several limitations in our study that need to be acknowledged. First, nomograms were established using retrospective data, which is associated with potential selection and information bias. After all, it’s easy to answer “no” or “unknown” than to recall the real solution. Therefore, prospective studies should be encouraged for further research. Second, some potentially important factors were not included in the SEER database, such as the human immunodeficiency virus and Epstein–Barr virus detection, history of alcohol and tobacco consumption, LDH, Eastern Cooperative Oncology Group and ultrasonic examination, which suggests that future studies of H&NNHL need to include these meaningful factors. Third, the SEER database does not separate “no” and “unknown” status. After all, there exists difference between them. In addition, more detailed information such as extent of radiation protocols used, the specific chemotherapy regimens and usage of new treatment strategies were not included and are expected in future researches. Fourth, the AJCC staging system has reached the 8th edition, but our research was still based on the 7th edition because it’s the latest record in the SEER database. It’s also a limitation and need to be improved as the database is updated. Finally, external validation of the nomogram was not performed, using only internal validation may lead to overfitting of new models. Further researches of multi-centered, large samples, diverse clinical, biological, and histopathologic factors are expected to measure the suitability of our nomograms among general population and confirm its clinical application value. Despite such inevitable limitations, we believe that our results, to a certain extent, have important implications in examining H&NNHL epidemiology and prognostic indicators of survival.

## 5. Conclusion

This is the first research to establish comprehensive nomograms for predicting OS and CSS in patients with H&NNHL at 3-, 5- and 8-year. The validation of the models demonstrated good performance. With the reference of the nomograms, physicians could appropriately determine the clinical treatment options and specifically predict the survival probability at specified time intervals for each patient. However, external validation of the nomogram should be added in future research.

## Acknowledgments

We thank all staff and patients of the SEER program. We are especially grateful to Professor Jun Lyu for his technical and statistical support in this study.

## Author contributions

**Conceptualization:** Jing Peng, Bin Zhang.

Data curation: Yucheng Liu.

Formal analysis: Jing Peng.

Investigation: Jing Peng, Jianming Chen.

Methodology: Jun Lyu.

Software: Jing Peng, Yucheng Liu.

Supervision: Jun Lyu, Bin Zhang.

Validation: Jing Peng, Jianming Chen.

Writing – original draft: Jing Peng.

Writing – review & editing: Jing Peng, Jianming Chen, Yucheng Liu, Jun Lyu, Bin Zhang.
